# Assessing the effect of adverse economic events on severity of hunger among food pantry clients

**DOI:** 10.3389/fpubh.2023.1286094

**Published:** 2023-10-31

**Authors:** Candice Bangham, Rachel M. Zack, Eva Nelson, Xinyang Liu, Alyson Codner, Jacqueline Milton Hicks, Jacey A. Greece

**Affiliations:** ^1^Department of Community Health Sciences, Boston University School of Public Health, Boston, MA, United States; ^2^Department of Family Medicine and Community Health, University of Massachusetts Chan Medical School, Worcester, MA, United States; ^3^Department of Biostatistics, Boston University School of Public Health, Boston, MA, United States

**Keywords:** hunger, food insecurity, adverse economic events, food pantry, economic instability

## Abstract

This study assessed relationship between adverse economic events (AEE) and hunger level (i.e., little to no, moderate, severe). A cross-sectional survey was conducted from June to August 2018 in 10 food pantries with 616 food pantry users. Hunger level was assessed by the Household Hunger Scale. AEE were evaluated over the past 3 months. Participants (60.55%) experienced unexpected or increased medical expenses (17.69%), job loss (13.64%), pay reduction (11.85%), and death of a family member (9.09%). Pay reduction (OR = 1.87, 95% CI: 1.12, 3.14) and increased debt (OR = 2.71, 95% CI: 1.92, 3.84) were associated with moderate hunger; death of a family member (OR = 2.43, 95% CI: 1.21, 4.90), pay reduction (OR = 2.95, 95% CI: 1.24, 7.04), and increased debt (OR = 3.46, 95% CI: 1.98, 6.04) were associated with severe hunger. Awareness of AEE can inform public health programs and policies for people in need of additional resources, which is essential in times of increased economic instability.

## 1. Introduction

Adverse economic events, including job loss, changes in family structure, and poor health can frequently lead to economic instability (1–4). The COVID-19 pandemic has prompted many Americans to experience increased adverse economic events (5, 6) in particular job loss (7), income loss (8), and emotional strain and financial worry (9), which all have the potential to increase risk of food insecurity (6). While national efforts have been underway for some time to alleviate the impact of adverse economic events on well-being, particularly related to housing and food access, a more thorough understanding of the economic risk factors that contribute to food insecurity allows for more targeted policy and program efforts, particularly in times of emergency that require rapid response.

Food insecurity, which is defined as having limited access to adequate food due to a lack of money or other resources (6), is categorized into 4 levels – very low food security (“at times during the year, eating patterns of one or more household members were disrupted and food intake reduced because the household lacked money and other resources for food”), low food security (“households reduced the quality, variety, and desirability of their diets, but the quantity of food intake and normal eating patterns were not substantially” disrupted), marginal food security (“households had problems at times, or anxiety about, accessing adequate food, but the quality, variety, and quantity of their food intake were not substantially reduced”), and high food security (“households had no problems, or anxiety about, consistently accessing adequate food”) (10, 11). Hunger is defined as a physical feeling of discomfort due to lack of food intake (12). While food insecurity and hunger are distinct concepts, they are closely related; some may feel hungry because they took too long to eat their meal, and others cannot fulfil their hunger feeling because they do not have food to eat due to financial constraints (12).

Food assistance programs such as food pantries provide food to help relieve hunger in populations that are in need. This results in allowing people access to resources to be better prepared to address the root causes of food insecurity. In 2020, 10.5% of U.S. households were food insecure and 3.9% experienced very low food security (13). Food insecurity is associated with a higher prevalence of chronic diseases (14–17) and is associated with a lower diet quality in people across the lifespan (18, 19) further contributing to the detrimental effects of food insecurity on long-term health outcomes. Addressing food insecurity through policy efforts and targeted programs could result in reduced costs to the larger health care system (20). There are a number of U.S. federal food assistance programs that target low-income populations (21, 22) and range in coverage from food assistance programs [i.e., Supplemental Nutrition Assistance Program (SNAP), Special Supplemental Nutrition Program for Women, Infants, and Children (WIC)] to child nutrition programs [i.e., School Breakfast Program (SBP), National School Lunch Program (NSLP), Summer Food Service Program (SFSP)] to food distribution programs [i.e., Commodity Supplemental Food Program (CSFP), The Emergency Food Assistance Program (TEFAP)]. While these programs offer huge benefit to populations experiencing food insecurity there is room for improvement and expansion (23), particularly at a time when shifting environmental conditions have impacted the existence and severity of food insecurity for certain populations (24).

Recent research indicates that certain sociodemographic determinants are associated with hunger in food pantry users such as marital status (being single, divorced, or separated): education (having less than high school education); work status (working part-time, unemployed, or retired); and, income (earning less than $1,000 per month) (25). Research is limited, however, on the individual and joint effects of specific types of adverse economic events on the existence and severity of food insecurity and hunger, such as unemployment, increased medical expenses, eviction, and experiencing the death of a family member. As such, this study, resulting from a broader examination of hunger in food pantry clients (25), aimed to understand which aspects of adverse economic events were most strongly associated with hunger among food pantry users in Massachusetts in 2018 and the extent to which these events affected the severity of hunger. In Massachusetts, food insecurity increased by 58% from 19% in 2019 pre-pandemic to 30% in 2020 during the start of the pandemic (26). This marked increase allows an examination of a state that would benefit from broader recommendations.

Although this study occurred pre-pandemic, many of the adverse economic events investigated during this study, occurred with increased frequency during the COVID-19 pandemic. Some adverse economic events, including job loss, increased medical expenses, and eviction, were exacerbated especially for populations already utilizing resources (13), such as those accessing food pantries, but in need of additional supports. The examination of adverse economic events and hunger, particularly in a population already struggling, helps to understand not only these relationships in non-pandemic times, but also allows exploration of effects of changes in economic events during pandemics or other emergency situations such as natural disasters (27) that require prioritization of public health efforts, re-allocation of resources, re-examination of policies, and targeted environmental approaches. Accordingly, the goal of this study was to examine the relationship between adverse economic events and hunger to gain a better understanding of the types of adverse economic events that most affect hunger and in populations already accessing supports but who are in need of more.

## 2. Methods

### 2.1. Participants and recruitment

This study, described elsewhere (25), conducted in partnership with The Greater Boston Food Bank (GBFB), recruited food pantry users visiting one of 10 selected food pantries between June 2018 and August 2018 to complete a survey. The 10 food pantries were selected based on high food pantry user volume, which was defined as serving at least 1,000 households per month in 2017. Study participants were required to: (1) be at least 18 years old or older; (2) be mentally and physically capable of completing the entire survey (as evidenced by their acknowledgement and participation in the informed consent process); (3) speak English or Spanish; and (4) be not planning on moving within the next three months. Recruitment occurred at each food pantry on multiple days and times of the week in an effort to capture a more representative sample of clients who visit that food pantry. Of the 1,444 participants that met these criteria on the days of recruitment at the food pantries occurred, 825 (57.1%) agreed to participate: reasons for refusal included lack of time; being in a rush; not speaking English or Spanish; and not understanding the study. The majority of respondents were not first-time food pantry users; they reported visiting a food pantry within the past 30 days (this was not part of the inclusion criteria of the study).

The 15-min survey was administered at the food pantry. Participants chose whether to complete the survey via self-administration on an iPad tablet (34.1%) or interview administration (65.9%) and provided oral informed consent. A majority (79.0%) of surveys were completed in English with 21.0% in Spanish. Participants received a $10 gift card as compensation for their time. This study was approved by Boston University’s Institutional Review Board (IRB # H-37567) as an exempt study with oral consent.

### 2.2. Measures

Hunger level, the outcome, was measured using a modified version of the validated Household Hunger Scale (HHS). This scale has been previously used for hunger monitoring and evaluation (28–31) and was used in this study as a proxy for the more traditional food insecurity assessments. The HHS was chosen for this study given it measures insufficient food quantity, is efficient in the food pantry setting, has been validated for use in a wide variety of cultures, and is appropriate for a population with a high level of food insecurity (25). Given the logistical and time considerations of conducting this study in a fast-paced food pantry setting, the HHS was modified for practical use in this study limiting the validity of the instrument. The modified HHS is composed of the following questions: (1) “In the past 30 days, how often was there ever no food to eat of any kind in your house because of lack of resources to get food?”; (2) “In the past 30 days, how often did you or any household member go to sleep at night hungry because there was not enough food?”; and (3) “In the past 30 days, how often did you or any household member go a whole day and night without eating anything at all because there was not enough food?.” Question response options include never (0 times), rarely (1–2 times), sometimes (3–10 times), and often (10+ times). Response options of “sometimes” and “often” were scored with 2, “rarely” was scored with 1, and “never” was scored 0 for each question (28–31). Per HHS protocol, the scores were then summed (range score 0 to 6) to create a hunger indicator score that was then categorized into ordinal and binary hunger variables. Ordinal hunger was defined as: little to no hunger (score = 0,1); moderate hunger (score = 2–3); and, severe hunger (score = 4–6). The binary hunger variable was defined as presence of moderate or severe hunger (score ≥ 2).

The exposure, adverse economic events (5), was evaluated by asking participants to select from a list the adverse economic event they or members of their household had experienced in the past 3 months. Participants could select all that apply from the following 10 options (variable labels in tables are provided in parentheses): (1) experienced significant (as determined subjectively by the respondent) out-of-pocket medical expenses (medical expenses); (2) lost a job (job loss); (3) had work hours and/or pay reduced (pay reduction); (4) were divorced (divorce); (5) received a foreclosure or eviction notice (eviction); (6) experienced the death of primary breadwinner or other family member (death of family member); (7) had loan repayment or interest/late fees from loans (debt); (8) had home repairs and increased cost of utilities (home-related expenses); (9) incurred legal expenses (legal expenses); and (10) other with a write-in option. Written responses for “other” were coded and recategorized into existing or new hardship categories (Table 1). Adverse economic events were quantified in three ways: (1) a binary variable defined as experience of any adverse economic events (i.e., at least one event reported); (2) an ordinal variable defined as total number of adverse economic events experienced (range of 0 to 6 with subsequent categories of none, 1 event, or 2 or more events); and (3) 10 binary variables defined as experience of each specific adverse economic events.

**Table 1:** Hunger Category by adverse economic event adjusted for food pantry and covariates, food pantry users in 10 food pantries in eastern Massachusetts, June 2018 – August 2018, *n* = 616.

	Moderate hunger^a^, *n* = 124		Severe hunger^a^, *n* = 121	
	Odds ratio (95% CI)	*p*-value^b^	Odds ratio (95% CI)	*p*-value^b^
Experience of adverse economic event^c^				
No	Ref		Ref	
Yes	2.03 (1.07, 3.85)	0.03	5.39 (2.78, 10.48)	<0.0001
Number of adverse economic events^d^				
0	Ref		Ref	
1	1.20 (0.83, 1.74)	0.33	0.74 (0.52, 1.06)	0.10
2+	2.09 (1.11, 3.92)	0.02	4.16 (2.39, 7.26)	<0.0001
Type of adverse economic events^e^				
Death of a family member	1.59 (0.75, 3.37)	0.23	2.43 (1.21, 4.90)	0.01
Divorce	1.31 (0.61, 2.83)	0.49	0.38 (0.02, 8.75)	0.55
Pay reduction	1.87 (1.12, 3.14)	0.02	2.95 (1.24, 7.04)	0.01
Legal expenses	0.21 (0.03, 1.67)	0.14	1.28 (0.66, 2.49)	0.46
Debt	2.71 (1.92, 3.84)	<0.0001	3.46 (1.98, 6.04)	<0.0001
Job loss	1.34 (0.80, 2.25)	0.26	1.77 (0.83, 3.79)	0.14
Home-related expenses	1.01 (0.34, 2.98)	0.9863	1.77 (0.60, 5.26)	0.3020
Eviction	4.19 (0.95, 18.50)	0.0587	4.19 (0.78, 22.51)	0.0947
Medical expenses	1.48 (0.75, 2.91)	0.2529	1.84 (0.84, 4.00)	0.1252
Other	1.56 (1.07, 2.27)	0.0218	1.64 (0.99, 2.72)	0.0541

a Hunger categories were defined as little to no hunger in the household (HHS score = 0–1), moderate hunger in the household (HHS score = 2–3), and severe hunger in the household (HHS 4–6) according to the HHS score. Both groups are compared to the no/little hunger group.

b Analyses were conducted using mixed effects modeling. The covariates included in the mixed-effects model are marital status, education status, age categories, income categories, seniors in the household, children in the household, race/ethnicity, occupation, and household size.

c Economic hardship was defined as experiencing at least one of the listed hardships in the past three months: medical expenses, job loss, reduced pay/h, divorce, home-related expenses, foreclosure/eviction notice, death of a family member or breadwinner, debt, legal expenses, or other hardship.

d Number of adverse economic events was determined based on the number selected by each participant.

e Types of adverse economic events were coded as separate variables.

Data collected on covariates included self-report of participant’s age, gender, educational attainment, race/ethnicity, marital status, occupational status, monthly household income, household size, and household composition [i.e., presence of children (<18 years old) or seniors (≥65 years old) in the household].

### 2.3. Data analysis

Participant characteristics were described overall and by hunger category with frequencies and percentages; Pearson’s chi-square test of independence were used to test for differences in characteristics by hunger category. Mixed effects models were employed because demographics of pantry users differed greatly by food pantry site, specifically by educational attainment level, race, and age. These models adjusted for food pantry site as a random effect while all other covariates were controlled for as fixed effects (25). Multivariable mixed effects models were used to estimate associations between economic instability and hunger category. Separate models were run defining the exposure in slightly different ways: experience with; number; and type of adverse economic events. These models all controlled for food pantry site as a random effect and all other covariates as fixed effects and in accordance with the approaches previously described (25). Analyses confirmed that missing data occurred at random. Analyses were performed using SAS^®^ (SAS^®^ version 9.4, SAS Institute Inc., Cary, NC, United States, 2013) with level of significance at 0.05.

## 3. Results

### 3.1. Participant characteristics

Of the 616 participants, the majority were female (72.6%), aged 50 years or older (60.6%), did not have children (57.5%) or seniors (70%) in their household, were non-Hispanic Black (26.5%) or Hispanic (28.3%), had high school or some college education (60.1%), were unmarried (63.2%), lived in a household with 2 or more people (71.1%), did not work full-time (84.8%), and in households that earned less than $1,500 per month (72.4%) (Table 2). Hunger level, assessed by responses to the modified HHS for this study, differed by age, educational attainment, race/ethnicity, household size, marital status, seniors, number of children in the household, household income, occupation, and food pantry site but did not change by pantry user status (new or existing) (25). Over half of participants had experienced an adverse economic event in the past 3 months (60.6%) with nearly one-quarter (23.4%) experiencing 2 or more instabilities. The most common adverse economic events were unexpected or increase in medical expenses (17.7%), job loss (13.7%), and reduction in pay (11.9%). Adverse economic events were more common in those with higher levels of hunger (51.8%) in participants with little to no hunger, 65.3% in participants with moderate hunger, 82.6% in participants with severe hunger.

**Table 2:** Hunger study participant characteristics by hunger level, food pantry users in 10 food pantries in eastern Massachusetts, June 2018 – August 2018, *n* = 616.

Variable	Overall (*N* = 616) *n* (%)	Little to no Hunger^a^ (*N* = 371) *n* (%)	Moderate Hunger^a^ (*N* = 124) *n* (%)	Severe Hunger^a^ (*N* = 121) *n* (%)	*p*-value^b^
Age, years^c^					
18 – < 30	50 (8.12%)	19 (5.12%)	14 (11.29%)	17 (14.05%)	0.0018
30 – < 40	88 (14.29%)	53 (14.29%)	18 (14.52%)	17 (14.05%)	
40 – < 50	105 (17.05%)	58 (15.63%)	22 (17.74%)	25 (20.66%)	
50 – < 60	178 (28.90%)	100 (26.95%)	38 (30.65%)	40 (33.06%)	
60 – < 65	70 (11.36%)	47 (12.67%)	14 (11.29%)	9 (7.44%)	
≥ 65	125 (20.29%)	94 (25.34%)	18 (14.52%)	13 (10.74%)	
Sex					
Female	447 (72.56%)	268 (72.24%)	99 (79.84%)	80 (66.12%)	0.0538
Male	169 (27.44%)	103 (27.76%)	25 (20.16%)	41 (33.88%)	
Educational attainment					
Less than high school	153 (24.84%)	85 (22.91%)	38 (30.65%)	30 (24.79%)	0.0325
High school or some college	370 (60.06%)	217 (58.49%)	74 (59.68%)	79 (65.29%)	
College graduate (4 years) or more	93 (15.10%)	70 (18.60%)	12 (9.68%)	12 (9.92%)	
Race/ethnicity					
Non-Hispanic White	230 (37.34%)	156 (42.05%)	36 (29.03%)	35 (28.93%)	0.0707
Non-Hispanic Black	163 (26.46%)	90 (24.26%)	38 (30.65%)	42 (34.71%)	
Non-Hispanic other	49 (7.95%)	23 (6.20%)	12 (9.68%)	27 (22.31%)	
Hispanic	174 (28.25%)	102 (27.49%)	38 (30.65%)	17 (14.05%)	
Household size^d^					
0–1 people	178 (28.90%)	117 (31.54%)	26 (20.97%)	35 (28.93%)	0.0398
2–3 people	235 (38.15%)	147 (39.62%)	46 (36.10%)	42 (34.71%)	
4–5 people	145 (23.54%)	79 (21.29%)	39 (31.45%)	27 (22.31%)	
≥ 5 people	58 (9.42%)	28 (7.55%)	13 (10.48%)	17 (14.05%)	
Marital status					
Single, never married	219 (35.55%)	110 (29.65%)	52 (41.94%)	57 (47.11%)	0.0018
Married, living with partner	227 (36.85%)	142 (38.27%)	45 (36.29%)	40 (33.06%)	
Separated, divorced, or widowed	170 (27.60%)	119 (32.08%)	27 (21.77%)	24(19.83%)	
Senior (≥ 65 years old) in household^e^	185 (30.03%)	127 (34.23%)	343(27.61%)	25 (20.66%)	0.0133
Child (<18 years old) in household^e^	262 (42.53%)	147 (39.30%)	66 (52.80%)	52 (42.98%)	0.0338
Monthly income^f^					
Less than $500	112 (18.18%)	61 (16.44%)	24 (19.35%)	27 (22.31%)	0.0067
$500 to $999	183 (29.71%)	93 (25.07%)	49 (39.52%)	41 (33.88%)	
$1,000 to $1499	151 (24.51%)	98 (26.15%)	24 (19.35%)	30 (24.79%)	
$1500 to $1999	89 (14.45%)	58 (15.63%)	16 (12.90%)	15 (12.40%)	
$2000 or more	81 (13.15%)	62 (16.71%)	11 (8.87%)	8 (6.61%)	
Occupation					
Disabled	152 (24.68%)	84 (22.64%)	31 (25.00%)	37 (30.58%)	0.0218
Homemaker	50 (8.12%)	33 (8.89%)	11 (8.87%)	6 (4.96%)	
Other	9 (1.46%)	4 (1.08%)	3 (2.42%)	2 (1.65%)	
Retired	94 (15.26%)	71 (19.14%)	15 (12.10%)	8 (6.61%)	
Unemployed	96 (15.58%)	52 (14.02%)	21 (16.94%)	23 (19.01%)	
Working full time (> = 35 h/week)	94 (15.26%)	63 (16.98%)	12 (9.68%)	19 (15.70%)	
Working part time (<35 h/week)	121 (19.64%)	64 (17.25%)	31 (25.00%)	26 (21.49%)	
Food pantry site					
Pantry 1	126 (20.45%)	69 (18.60%)	24 (19.35%)	33 (27.27%)	0.0337
Pantry 2	21 (3.41%)	15 (4.04%)	4 (3.23%)	2 (1.65%)	
Pantry 3	25 (4.06%)	13 (3.50%)	7 (5.65%)	5 (4.13%)	
Pantry 4	98 (15.91%)	55 (14.82%)	23 (18.55%)	20 (16.53%)	
Pantry 5	18 (2.92%)	8 (2.16%)	5 (4.03%)	5 (4.13%)	
Pantry 6	18 (2.92%)	11 (2.96%)	6 (4.84%)	1 (0.83%)	
Pantry 7	38 (6.17%)	18 (4.85%)	10 (8.06%)	10 (8.26%)	
Pantry 8	215 (34.90%)	151 (40.70%)	30 (24.19%)	34 (28.10%)	
Pantry 9	51 (8.28%)	30 (8.09%)	13 (10.48%)	8 (6.61%)	
Pantry 10	6 (0.97%)	1 (0.27%)	2 (1.61%)	3 (2.48%)	
Experience of adverse economic events^g^					
Yes	373 (60.55%)	192 (51.75%)	81 (65.32%)	100 (82.64%)	<0.0001
No	243 (39.45%)	179 (48.25%)	43 (34.68%)	21 (17.36%)	
Number of adverse economic events					
0	264 (42.84%)	188 (50.67%)	50 (40.32%)	26 (21.49%)	<0.0001
1	208 (33.77%)	110 (29.65%)	44 (35.48%)	54 (44.63%)	
2 or more	144 (23.38%)	73 (19.68%)	30 (24.19%)	41 (33.88%)	
Type of adverse economic event^h^^,^^i^					
Death of family member	56 (9.09%)	24 (6.47%)	13 (10.48%)	19 (15.70%)	
Divorce	11 (1.79%)	7 (1.89%)	3 (2.42%)	1 (0.83%)	
Pay reduction	73 (11.85%)	32 (8.63%)	17 (13.71%)	24 (19.83%)	
Legal expenses	26 (4.22%)	17 (4.58%)	2 (1.61%)	7 (5.79%)	
Debt	61 (9.90%)	27 (7.28%)	16 (12.90%)	18 (14.88%)	
Job Loss	84 (13.64%)	40 (10.78%)	19 (15.32%)	25 (20.66%)	
Home-related expenses	28 (4.55%)	17 (4.58%)	4 (3.23%)	7 (5.79%)	
Eviction	31 (5.03%)	8 (2.16%)	10 (8.06%)	13 (10.74%)	
Medical expenses	109 (17.69%)	62 (16.71%)	21 (17.94%)	26 (21.49%)	
Other	117 (18.99%)	66 (17.79%)	26 (20.97%)	25 (20.66%)	

a Hunger categories were defined as little to no hunger in the household (HHS score = 0–1), moderate hunger in the household (HHS score = 2–3), and severe hunger in the household (HHS 4–6) according to the HHS score.

b Analyses were conducted using frequencies and Pearson’s chi-square statistical test significance = 0.05.

c Age categories were created based on pre-established age definitions from the US Census.

d Household size categories were created based on the open-ended responses of number of people in household.

e Household composition for both children and seniors in the household were defined as at least one or more in the household.

f Income categories were created based on open-ended responses for annual/monthly income.

g Adverse Economic Events was defined as experiencing at least one of the listed events in the past three months: medical expenses, job loss, reduced pay/h, divorce, home-related expenses, foreclosure/eviction notice, death of a family member or breadwinner, debt, legal expenses, or another event.

h This was a select more than one adverse economic event and therefore the probability may exceed 100%.

i Types of adverse economic events were coded as separate variables and therefore no tests of statistical significance are conducted on this overall variable but are conducted in later tables.

### 3.2. Multivariable mixed effect models

The results of multivariable mixed effect models examining the effect of adverse economic events on hunger level, adjusted for the food pantry attended, marital status, education status, age categories, income categories, seniors in the household, children in the household, race/ethnicity, occupation, and household size are shown in Table 1. Experience and number of adverse economic events were associated with higher odds of both moderate and severe hunger with severe hunger having higher odds than moderate hunger. Experience of any adverse economic event, compared to none, was associated with higher odds of moderate hunger (OR = 2.03, 95% CI: 1.07, 3.85) and severe hunger (OR = 5.39, 95% CI: 2.78, 10.48). Food pantry users that had 2 or more adverse economic events had higher odds of moderate hunger and severe hunger compared to having no adverse economic events (OR = 2.09, 95% CI: 1.11, 3.92 and OR = 4.16, 95% CI: 2.39, 7.26, respectively).

Reduction in pay and experiencing an increase in debt were both significantly associated with higher odds of moderate and severe hunger (reduction in pay – OR = 1.87, 95% CI: 1.11, 3.14 and OR = 2.95, 95% CI: 1.24, 7.04, respectively and debt – OR = 2.71, 95% CI: 1.92, 3.84 and OR = 3.46, 95% CI: 1.98, 6.04, respectively), with severe hunger having higher odds than moderate hunger for both types of instabilities. Death of a family member was associated with higher odds of severe hunger (OR = 2.44, 95% CI: 1.21, 4.90).

## 4. Discussion

This study documents that among food pantry users, experience with and number of certain adverse economic events resulted in increased odds of both moderate and severe hunger. In particular, experience with debt, reduction in pay, and eviction were significantly associated with moderate and severe hunger. Death of a family member was also significantly associated with severe hunger. Food pantry users that experienced two or more adverse economic events compared to no adverse economic events had significantly higher odds for moderate and severe hunger suggesting that compounded adverse economic events results in increased vulnerability to hunger. The most common economic household instabilities across all hunger categories (low, moderate, and severe) were unexpected or increased medical expenses, job loss, and reduction in pay in the past 3 months.

The findings of this study are consistent with the literature that shows adverse economic events impact food insecurity (32–34). Measures are in place, such as government assistance programs and economic relief payments, to address specific adverse economic events (6, 33, 35) and even with additional support during the pandemic, food insecurity and adverse economic events persist (6). The American Rescue Plan was enacted in March 2021 to address the hardships faced by many Americans as a result of the pandemic, which resulted in a 5% decline in the number of adults in the U.S. who reported not having enough to eat in the past 7 days in August 2021 (6). As these benefits expired, so did the relief Americans experienced (6) even though the economic instabilities or the impacts of them remain for many.

While this data was collected prior to the pandemic, adverse economic events highlighted in the findings of this study, have been exacerbated during the pandemic (13). For example, the unemployment rate in the U.S. increased from 4.4% in 2019 to 14.7% in April of 2020, during the height of the pandemic (36). While some lost work due to the economic repercussions of the pandemic, work status was directly impacted by the pandemic for some who contracted the virus; contracting the virus meant they were often unable to work. Those who suffered from job and income loss due to the pandemic had a harder time affording food for their households (7). In addition, loss of a family member also resulted in extra hardship. People who lost a household member due to COVID-19 may have lost a primary source of income, which led to further adverse economic events and impacted food security (37) including for children who are at significantly greater risk for food insecurity and Adverse Childhood Experiences (37) when living in households with lower incomes than in households with higher income (38).

Given the associations found in this study and in particular the types and amount of adverse economic events that are associated with hunger, we can expect that the increased prevalence of adverse economic events experienced during the pandemic can lead to substantial increases in hunger and food insecurity, particularly in populations already accessing services and supports for hunger such as food pantry clients. This is important in considering efforts to address hunger and food insecurity in populations already at-risk and specifically during times that increase disadvantageous conditions for these populations such as during a pandemic, natural disasters, and other emergency situations (27).

There have been efforts to address adverse economic events throughout and since the pandemic. For example, the number of adult renters who reported that they were not caught up on rent declined after the disbursement of emergency aid funded via the December 2020 relief package and American Rescue Plan, however many adult renters still faced challenges in paying rent due to accumulated debt from job disruption and late fees associated with inability to pay rent for multiple months (6). Specifically, people of color and households with children reported higher rates of rent hardship (i.e., not being caught up on rent, throughout 2020 and 2021) (6).

Job loss and unemployment skyrocketed during the pandemic to rates not previously seen since the Great Depression, with job losses concentrated in the lowest paying industries (3). Our findings indicate that, among food pantry users, job loss significantly increased the odds of being severely hungry even before the repercussions of the pandemic. During the pandemic, the country responded by funding new or expanding existing programs to reduce the financial burden to families and address hunger. For example, the Pandemic-Electronic Benefit Transfer (P-EBT) program provided funding for states to allocate resources directly to households with children who lost access to school meal programs during COVID-19 in an effort to reduce child food insecurity (39, 40). While these types of supports emerged due to the pandemic, this research indicates the necessity of continuation of them given associations that existed even before the pandemic.

Increasing the accessibility of and eligibility for long-standing federal nutrition assistance programs, such as SNAP, can also help mitigate potential hunger impacts of adverse economic events. States often have flexibility in how they implement federal programs, which can allow for increased accessibility and flexibility of federal nutrition assistance programs. For example, USDA regulations include asset limits for SNAP, which means that a low-income household might not be eligible for SNAP due to having assets. One method in which states can increase SNAP eligibility is through removing asset limits through a policy called broad-based eligibility (41). Additional states could remove asset limits to increase SNAP availability for low-income households who may have a small amount of assets, but still be only one adverse economic event away from experiencing hunger.

While prior to and during the pandemic safety net programs and policies existed to alleviate food insecurity, in order to continually address adverse economic events and hunger, multiple interventions are needed to address the issue of food insecurity particularly on those who have faced, and continue to face these challenges (40). While our study did not find an association between home expenses and food insecurity, there was an association between factors (i.e., increased debt, reduction in pay, and eviction) that could affect someone’s ability to retain their housing. Research has shown that some government programs to address adverse economic events have reduced food insecurity during the pandemic (42). For example, the expanded Child Tax Credits, beginning in July of 2021, reduced household food insufficiency by 26% (42). However, this was a temporary solution. Community Information Exchanges (CIE), which compile information for many community organizations that address different, but interconnected, needs have also shown to be successful in addressing specific social determinants that impact food insecurity, housing instability, and other adverse economic events (32). Accordingly, efforts should focus on populations that use food assistance and who have compounded hardship due to experience of these adverse economic events.

A strength of this study was the ability to assess the population on hunger status using the modified Household Hunger Scale (HHS), which allowed for the ability to efficiently quantify hunger levels of food pantry clients from a wide variety of cultures (28–31) though also had some limitations as described below. The large sample size of food pantry users speaking English or Spanish from 10 food pantries allowed for examination of a population already accessing services to address hunger pre-pandemic. Participants in the study experienced a number of adverse economic events allowing for an in-depth analysis of the type and cumulative number of instabilities experienced. This study was conducted before the pandemic, which helps to see the existing associations between hunger and hardship absent before an event that caused exacerbated economic stressors.

Despite the strengths of this study, limitations should be considered. First, this study was conducted in eastern Massachusetts, which is a narrow geographic area, so the results are not necessarily representative of the U.S. population. The findings, however, can inform approaches for food pantry clients who would benefit from enhanced resources. Second, there may be differential misclassification as individuals who were more likely to report adverse economic events may have also been more likely to report experiencing hunger. Third, the sample represents the food pantry clients who were present at the pantry on the day of recruitment and may not represent all pantry clients at that particular food pantry, although, we recruited at the food pantries on multiple days and times of each week. Fourth, the scale used to assess hunger is a 3-item modification of a validated 6-item scale and was used to feasibly administer a survey in a fast-paced food pantry setting to encourage greater participation and response. While not ideal we were able to obtain high participation that would have otherwise been difficult with a longer survey. Still, results should be interpreted with caution given this scale was modified and was not validated and social desirability is likely to have influenced responses. Other researchers conducting similar research should consider this against the logistical constraints of a longer but validated survey in dynamic research settings. Finally, we were unable to control for other factors that may impact adverse economic events and hunger such as housing situation (i.e., temporary versus permanent) and homelessness.

Findings from this study can inform considerations for expansion and sustainability of efforts to address food insecurity, particularly those enacted during the pandemic. Adverse economic events such as debt, reduction in pay, eviction, increased medical expenses, job loss, and death of a family member were exacerbated during the pandemic with an increase in government assistance to address them. As public health considers areas for intervention in policy development and program expansion for populations facing hunger and food insecurity, these data support consideration of the types and quantity of adverse economic events most affecting populations already in need of resources to ensure the root causes of hunger are addressed by ongoing, sustained efforts and appropriate allocation of resources and prioritization of planning. Further investigation of the impact of adverse economic events on use of food assistance programs (e.g., food pantries, SNAP), mental health disorders, and other adverse health outcomes could be beneficial to understanding the full cost of the economic repercussions of the pandemic. Additionally, future research to understand whether adverse economic events disproportionately increase hunger among certain demographic groups (e.g., race/ethnicity, immigration status, gender, households with children, seniors, etc.) is important when ensuring that programs and policies designed to address adverse economic events work to diminish, rather than increase, inequities (43).

## Data availability statement

The raw data supporting the conclusions of this article will be made available by the authors, without undue reservation.

## Ethics statement

The studies involving humans were approved by Boston University’s Institutional Review Board (IRB #H-37567) as an exempt study with oral consent. The studies were conducted in accordance with the local legislation and institutional requirements. Written informed consent for participation was not required from the participants or the participants’ legal guardians/next of kin because oral informed consent was obtained per IRB guidance.

## Author contributions

CB: Data curation, Formal analysis, Writing – original draft, Writing – review & editing. RZ: Conceptualization, Data curation, Formal analysis, Investigation, Methodology, Supervision, Writing – review & editing. EN: Writing – original draft, Writing – review & editing. XL: Data curation, Formal analysis, Methodology, Writing – review & editing. AC: Data curation, Formal analysis, Methodology, Writing – original draft, Writing – review & editing. JH: Conceptualization, Formal analysis, Methodology, Resources, Supervision, Writing – review & editing. JG: Conceptualization, Data curation, Formal analysis, Investigation, Methodology, Project administration, Resources, Supervision, Writing – original draft, Writing – review & editing.
